# Coreolanceolins A–E, New Flavanones from the Flowers of *Coreopsis lanceolate,* and Their Antioxidant and Anti-Inflammatory Effects

**DOI:** 10.3390/antiox9060539

**Published:** 2020-06-19

**Authors:** Hyoung-Geun Kim, Young Sung Jung, Seon Min Oh, Hyun-Ji Oh, Jung-Hwan Ko, Dae-Ok Kim, Se Chan Kang, Yeong-Geun Lee, Dae Young Lee, Nam-In Baek

**Affiliations:** 1 Department of Oriental Medicinal Biotechnology, Graduate School of Biotechnology, Kyung Hee University, Yongin 17104, Korea; zwang05@khu.ac.kr (H.-G.K.); seonmin88@khu.ac.kr (S.M.O.); chkd12@naver.com (H.-J.O.); hwann92@naver.com (J.-H.K.); sckang@khu.ac.kr (S.C.K.); lyg629@nate.com (Y.-G.L.); 2 Department of Food Science and Biotechnology, Kyung Hee University, Yongin 17104, Korea; chembio@khu.ac.kr (Y.S.J.); DOKIM05@khu.ac.kr (D.-O.K.); 3 Department of Herbal Crop Research, National Institute of Horticultural and Herbal Science, RDA, Eumseong 27709, Korea

**Keywords:** antioxidant capacity, anti-inflammation, *Coreopsis lanceolata*, coreolanceolin, flower

## Abstract

(1) Background: Many flavonoids derived from natural sources have been reported to exhibit antioxidant and anti-inflammatory effects. Our preliminary study suggested that *Coreopsis lanceolata* flowers (CLFs) include high flavonoid content; (2) Methods: CLFs were extracted in 80% (v/v) aqueous methanol and fractionated into ethyl acetate, *n*-butanol, and water fractions. Repeated column chromatographies for the organic fractions led to the isolation of seven flavanones. Quantitative analysis of the flavanones was carried out using reversed-phase high-performance liquid chromatography. All flavanones were evaluated for their antioxidant and pro-inflammatory inhibition effects; (3) Results: Spectroscopic analyses revealed the chemical structure of five new flavanones, coreolanceolins A–E, and two known ones. The content of the seven flavanones in extracts were determined from 0.8 ± 0.1 to 38.8 ± 0.3 mg/g. All flavanones showed radical scavenging activities (respectively 104.3 ± 1.9 to 20.5 ± 0.3 mg vitamin C equivalents (VCE)/100 mg and 1278.6 ± 26.8 to 325.6 ± 0.2 mg VCE/100 mg) in the DPPH and 2,2′-Azino-bis(3-ethylbenzothiazoline-6-sulfonic acid) (ABTS) assays and recovery activities in Caco-2 (59.7 to 41.1%), RAW264.7 (87.8 to 56.0%), and PC-12 (100.5 to 69.9%) cells against reactive oxygen species. Furthermore, all flavanones suppressed nitric oxide production (99.5% to 37.3%) and reduced iNOS and COX-2 expression in lipopolysaccharide-treated RAW 264.7 cells; (4) Conclusions: Five new and two known flavanones were isolated from CLF, and most of them showed high antioxidant and pro-inflammatory inhibition effects.

## 1. Introduction

The *Coreopsis* genus is the phanerogam in the Asteraceae family. *Coreopsis* is commonly called “Calliopsis” or “tickseed.” The flowers are usually yellow or yellow-and-red bicolor with a toothed tip. The *Coreopsis* genus is widely distributed in North and South America, as well as Eastern Asia [1]. *Coreopsis lanceolata, C. drummondii*, and *C. tinctoria* are universal *Coreopsis* plants distributed all over Korea. *Coreopsis lanceolata* is the most common *Coreopsis* that is encountered in Korea and has bigger flowers than the other two species.

*Coreopsis lanceolata* is an herbaceous perennial plant that grows from 30 to 100 cm in length with lanceolate leaves. The flowers are obovate, 4–6 cm in diameter, have ends split like sawtooth, and bloom in summer [2]. Our previous study reported the isolation and identification of 12 chalcones from *C. lanceolata* flowers (CLFs), including seven new compounds, and their chemo-preventive effects against human colon cancer cells [3]. Biogenetically, chalcones are the immediate precursors of flavanones [4]. Therefore, it is predictable that CLF contain various flavanones. Furthermore, recent reports that CLF has antioxidant [5], anti-allergic [6], antibacterial [6], anti-leukemic [7], and nematocidal [8] effects, indicate that CLF also include a variety of antioxidant and anti-inflammatory flavonoids. Despite the reported pharmacologic activity of *C. lanceolata*, only one flavanone ((2*S*)-7,3′,4′-trihydroxy-8-methoxyflavanone) has been isolated from the its flowers [7]. In this study, we identified new flavanones with antioxidant and pro-inflammatory inhibition activities from CLF.

Oxidative stress (OS) in the human body is caused by overwhelmed reactive oxygen species (ROS), including superoxide (SO) and hydrogen peroxide, and it is related to a variety of apoptotic types of cell death such as neuronal cell death, intestinal epithelial cell damage, and macrophage immune injury. If ROS are not adequately removed, OS can cause the lipid peroxidation of the cellular membrane leading to inordinate cell death.

Macrophages play critical roles in the human immune system and mainly defend the body against external pathogens through phagocytosis and the expression of cytokines. During the immune response, nitric oxide (NO) and excessive cytokines are produced as mediators of inflammatory responses [9,10]. Inflammatory cytokines can accelerate the expression of ROS, thereby damaging the tissue in the body. In general, ROS are eliminated by antioxidant enzymes such as SO dismutase and antioxidant agents such as glutathione. However, if the inflammation is associated with a disease or a problem in metabolism such as chronic inflammation, type 2 diabetes, and rheumatoid arthritis, the body can fail to effectively remove the ROS. Furthermore, as the inflammatory reaction progresses, excessive ROS production can result in fatal damage to surrounding tissues [11,12]. Therefore, to prevent the ROS-mediated damage caused by disease-related inflammation, it is useful to take antioxidant supplements. However, synthetic antioxidants and anti-inflammatory agents can be toxic and carcinogenic and they can interfere with the metabolic and respiratory activities of cells. Therefore, alternative, natural antioxidants and anti-inflammatory agents without side effects need to be developed.

We evaluated the antioxidant and pro-inflammatory inhibition properties of flavanones which were isolated from CLF from various angles in vitro such as enteric epithelial Caco-2 cells, RAW 264.7 macrophage cells, and neuron PC-12cells. Furthermore, we investigated the correlation between pro-inflammatory inhibition and antioxidant activity in RAW 264.7 cells. Since the new flavanones found in this study have a diverse variation in their structural characteristics and potent antioxidant and pro-inflammatory inhibition activities, their successive study will lead to the development of safe and effective functional materials against metabolic disease.

## 2. Materials and Methods 

### 2.1. Plant Materials

CLFs were collected, identified, and stored by the same procedure as has been previously reported in the literature [3].

### 2.2. General Experimental Procedures

The materials and equipment used for the isolation and structure determination of the constituents are described in a previous study [3,13]. Antibodies against inducible nitric oxide synthase (iNOS) (sc-8310), cyclooxygenase-2 (COX-2) (sc-1747), and *β*-actin (#3700) were obtained from Santa Cruz Biotechnology (Santa Cruz, CA, USA) and Cell Signaling Technology (Beverly, MA, USA). The PC-12 and RAW 264.7 cell lines were purchased from the American Type Culture Collection (Manassas, VA, USA). The Caco-2 cell line was purchased from the Korean Cell Line Bank (Seoul, Republic of Korea).

### 2.3. Extraction and Isolation

**Figure 1:**
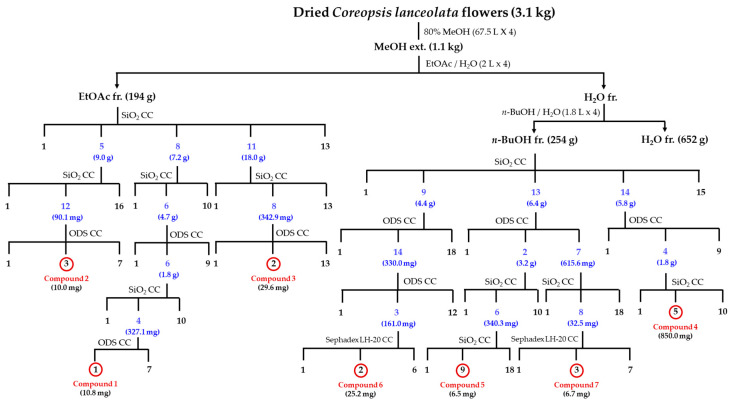
Isolation procedures of compound **1**–**7** from *Coreopsis lanceolata* flowers. fr.: fraction; CC: column chromatography; SiO_2_: silica gel; ODS: octadecyl silica gel.

CLFs (3.1 kg of dry weight (DW)) were extracted in 80% (v/v) aqueous methanol (MeOH) (67.5 L × 4) at ambient temperature for 24 h, filtered, and evaporated to yield a residue of 1.1 kg. The obtained MeOH extracts were suspended in water (H_2_O) (2 L) and then successively extracted with ethyl acetate (EtOAc) (2 L × 4) and *n*-butanol (BuOH) (1.8 L × 4). Each layer was concentrated under reduced pressure to obtain EtOAc (CLFE; 194 g), *n*-BuOH (CLFB; 254 g), and H_2_O (CLFW; 652 g) soluble fractions (frs). The isolation procedures of compounds 1–7 from CLF are presented in Figure 1 and Figure S1 (see the Supplementary Materials).

**8-Methoxybutin (1):** Red amorphous powder (CH_3_OH); ${\left[\alpha \right]}_{D}^{25}$ +21.9° (*c* 0.10, CH_3_OH); IR (CaF_2_ window) 3360, 1615, and 1590 cm^−1^; positive FAB-MS (FM) *m/z* 303 [M + H]^+^; ^1^H-NMR (400 MHz, CD_3_OD, δ_H_) (PMRM400) and ^13^C-NMR (100 MHz, CD_3_OD, δ_C_) (CMRM100) data were consistent with those in the literature [14].

**Coreolanceolin A (7,3′-dihydroxy-8,5′-dimethoxyflavanone) (2)**: Red amorphous powder (CH_3_OH); ${\left[\alpha \right]}_{D}^{25}$ +21.8° (*c* 0.10, CH_3_OH); CD (*c* 0.2, MeOH): 335 (Δε +0.2), 290 (Δε −2.1); IR (CaF_2_ window) 3427, 1637, and 1579 cm^−1^; positive high resolution (HR)-FM *m/z* 317.1028 [M + H]^+^ (calcd for C_17_H_17_O_6_, 317.1025); PMRM400 and CMRM100 refer to Table 1 and Table 2. 

**Flavanomarein (3)**: Yellow amorphous powder (CH_3_OH); ${\left[\alpha \right]}_{D}^{25}$ −70.8° (*c* 0.10, CH_3_OH); IR (CaF_2_ window) 3362, 1609, and 1589 cm^−1^; positive FM *m/z* 451 [M + H]^+^; PMRM400 and CMRM100 data were consistent with those in literature [15].

**Coreolanceolin B (7,3′,4′-trihydroxy-8-methoxyflavanone 7-*O*-*β*-D-glucopyranoside) (4):** Red amorphous powder (CH_3_OH); ${\left[\alpha \right]}_{D}^{25}$ −71.0° (*c* 0.10, CH_3_OH); CD (*c* 0.2, MeOH): 399 (Δε +0.1), 290 (Δε −0.3); IR (CaF_2_ window) 3490, 1634, and 1580 cm^−1^; positive HR-FM *m/z* 465.1395 [M + H]^+^ (calcd for C_22_H_25_O_11_, 465.1396); ^1^H-NMR (400 MHz, pyridine-*d*_5_, δ_H_) and ^13^C-NMR (100 MHz, pyridine-*d*_5_, δ_C_) refer to Table 1 and Table 2.

**Coreolanceolin C (7,8,4′-trihydroxy-3′-methoxyflavanone 7-*O*-*β*-D-glucopyranoside) (5):** Red amorphous powder (CH_3_OH); ${\left[\alpha \right]}_{D}^{25}$ −70.7° (*c* 0.10, CH_3_OH); CD (*c* 0.2, MeOH): 387 (Δε +0.2), 287 (Δε −0.1); IR (CaF_2_ window) 3489, 1634, and 1580 cm^−1^; negative HR-FM *m/z* 463.1238 [M − H]^–^ (calcd for C_22_H_23_O_11_, 463.1240); PMRM400 and CMRM100 refer to Table 1 and Table 2.

**Coreolanceolin D (7,3′-dihydroxy-8,4′-dimethoxyflavanone 7-*O*-*β*-D-glucopyranoside) (6):** Red amorphous powder (CH_3_OH); ${\left[\alpha \right]}_{D}^{25}$ −70.4° (*c* 0.10, CH_3_OH); CD (*c* 0.2, MeOH): 386 (Δε +0.2), 274 (Δε −2.1); IR (CaF_2_ window) 3500, 1640, and 1590 cm^−1^; negative HR-FM *m/z* 477.1395 [M − H]^–^ (calcd for C_23_H_25_O_11_, 477.1396); PMRM400 and CMRM100 refer to Table 1 and Table 2.

**Coreolanceolin E (7,3′-dihydroxy-8,5′-dimethoxyflavanone 7-*O*-*β*-D-glucopyranoside) (7):** Red amorphous powder (CH_3_OH); ${\left[\alpha \right]}_{D}^{25}$ −69.9° (*c* 0.10, CH_3_OH); CD (*c* 0.2, MeOH): 391 (Δε +0.2), 277 (Δε −2.2); IR (CaF_2_ window) 3502, 1638, and 1588 cm^−1^; positive HR-FM *m/z* 501.1374 [M+Na]^+^ (calcd for C_23_H_26_O_11_Na, 501.1372); PMRM400 and CMRM100 refer to Table 1 and Table 2.

### 2.4. Quantitative Analysis of Compound ***1***–***7*** Using Reversed-Phase HPLC

Quantitative analysis of the flavonoids was performed using reversed-phase high-performance liquid chromatography (HPLC) (Alliance e2690; Waters Corp., Milford, MA, USA) with a C18 column (Poroshell 120 SB-C_18_; 120 Å, 2.7 μm, 4.6 × 150 mm; Agilent Technologies, Santa Clara, CA, USA). The column oven temperature was 40 °C, the sample injection volume was 5 μL, and the detection wavelength was set to 360 nm. Solvent A (H_2_O: formic acid = 99.9: 0.1, v/v) and solvent B (acetonitrile) were used in the mobile phase, and the flow rate was set to 0.8 mL/min. The solvent elution was graded as follows: 95% A/5% B at 0 min, 95% A/5% B at 1 min, 80% A/20% B at 3 min, 80% A/20% B at 8 min, 77% A/23% B at 10 min, 77% A/23% B at 13 min, 72% A/28% B at 15 min, 72% A/28% B at 20 min, 20% A/80% B at 22 min, 95% A/5% B at 24 min, and 95% A/5% B at 26 min. For the quantitative analysis of compound **1**–**7** isolated from the CLF extract, 1 mg of each flavanone was accurately weighed and dissolved in MeOH to obtain stock solutions with a concentration of 1.0 mg/mL. Calibration curves were developed for each standard with six different concentrations (100, 50, 25, 12.5, 6.25, and 3.125 µg/mL). A volume of 1 milligram obtained from the CLF extract was also accurately weighed and dissolved in 80% (v/v) aqueous MeOH to create stock solution with a concentration of 5.0 mg/mL. The quantitative analysis was repeated three times.

### 2.5. Antioxidant Activities 

#### 2.5.1. Free Radical Scavenging Activity

Antioxidant capacities were determined using the 2,2′-azino-bis(3-ethylbenzothiazoline-6-sulfonic acid) (ABTS) and 2,2-diphenyl-1-picrylhydrazyl (DPPH) radical scavenging assays [16,17]. Briefly, the ABTS radical solution was adjusted to an absorbance of 0.650 ± 0.020 at 734 nm. Reactions between the ABTS radical solution and the diluted compound **1**–**7** (satisfying the standard curve range) were allowed to proceed at 37 °C for 10 min, and decreases in the absorbance of the resulting solution were measured using a spectrophotometer (SPECTRONIC 200; Thermo Fisher Scientific Inc., Waltham, MA, USA). For the DPPH assay, the absorbance of DPPH radicals in 80% (v/v) aqueous methanol was set to 0.650 ± 0.020 at 517 nm. Reactions between the DPPH radical solution and the diluted compound **1**–**7** (satisfying the standard curve range) were allowed to proceed at ambient temperature for 30 min. Decreases in the absorbance of the resulting solution were monitored at 517 nm using a spectrophotometer (SPECTRONIC 200). Antioxidant capacities were expressed as mg vitamin C equivalent (VCE)/100 mg.

#### 2.5.2. Cell Culture and Cytotoxicity

For the cell culture, a complete medium (with the addition of 10% heat-inactivated fetal bovine serum (FBS), 100 units/mL penicillin, and 100 μg/mL of streptomycin) was used. RAW 264.7 and Caco-2 cells were cultured in complete Dulbecco’s modified Eagle’s medium (DMEM), and PC-12 cells were cultured in complete Roswell Park Memorial Institute (RPMI) 1640 medium. All cell lines were sub-cultured when approximately 90% of the distribution was in the culture dish. To determine the non-toxic maximal concentration of compound **1**–**7**, cytotoxicity was assessed using a 3-(4,5-dimethylthiazol-2-yl)-2,5-diphenyl-tetrazolium bromide (MTT) reduction assay [18]. PC-12, Caco-2, and RAW 264.7 cells were seeded at a density of 2 × 10^4^ cells/well in a 96-well plate in RPMI 1640 or DMEM medium containing FBS and left for 24 h. After removing the medium, cells were treated with a serum-free medium containing compound **1**–**7** at various concentrations. Following a 24 h incubation period, the medium was removed from each well. Then, MTT reagent was added, the plate was incubated for 3 h, and 50 μL of DMSO was added. The absorbance was measured using a microplate reader (Infinite M200; Tecan Austria GmbH, Grödig, Austria) at 570 nm (test wavelength) and 630 nm (reference wavelength). The cytotoxic effect was expressed as the percentage (%) of metabolically active cells relative to control cells cultured without test samples.

#### 2.5.3. Measurement of Intracellular OS

Intracellular OS levels were evaluated using 2′,7′-dichlorofluorescein diacetate (DCFH-DA) following previously reported methods [19]. Briefly, Caco-2, RAW 264.7 and PC-12 cells were seeded (2 × 10^4^ cells/well in a 96-well plate) in their respective complete media and incubated for 3 h in a humidified incubator with 5% CO_2_ at 37 °C. The cells were then treated with non-toxic concentrations (0.625–10 μg/mL) of compound **1**–**7**. After removing the supernatant, 50 μM DCFH-DA in phosphate-buffered saline (PBS) was added and incubated for 30 min, and then the cells were treated with 200 μM H_2_O_2_ for 30 min. Fluorescence was measured using a microplate reader (Infinite M200) with excitation at 485 nm and emission at 530 nm.

### 2.6. Pro-Inflammatory Inhibition Activity Assay 

#### 2.6.1. Determination of NO Production

NO produced by RAW 264.7 cells was determined using a method reported in the literature [3]. In brief, RAW 264.7 cells at a density of 4 × 10^5^ cells/well in a 96-well plate were pre-cultured for 24 h. The cells were then stimulated with 1 μg/mL of lipopolysaccharide (LPS) in the presence of samples for 24 h. A supernatant was obtained to evaluate the nitrite level using the Griess reagent system. The nitrite level was determined by measuring the absorbance at 540 nm with a microplate reader (Infinite M200). The nitrite concentration was extrapolated from the standard curve of sodium nitrite.

#### 2.6.2. Western Blot Analysis for Protein Expression

The intracellular content of pro-inflammatory enzymes was measured by Western blotting. RAW 264.7 cells at a density of 2 × 10^6^ cells/well were pre-cultured in a 6-well plate for 24 h and then stimulated with 1 μg/mL LPS and 1 ng/mL *β*-actin in the presence of samples for 24 h. Total cell extracts were obtained by a lysis buffer (50 mM Tris-HCl, pH 7.5; 150 mM NaCl; 1mM EDTA; 20 mM NaF; 0.5% NP-40; 1% Triton X-100) containing a protease inhibitor cocktail (GenDEPOT, Barker, TX, USA). The protein concentration was quantified using the Bradford assay. Cell extracts were loaded onto an 12% sodium dodecyl sulfate polyacrylamide gel and transferred to nitrocellulose membranes. The membranes were incubated with iNOS (1:500) and COX-2 (1:500) overnight at 4 °C. Subsequently, horseradish-peroxidase-conjugated anti-mouse or anti-rabbit secondary antibody was used for 1 h at ambient temperature. Blots were detected using EzWestLumi plus (ATTO, Tokyo, Japan) and analyzed using an EZ-Capture MG (ATTO). The band density was quantified using Image J software (Bogdan, 2001, Stuttgart, Baden-Wurttemberg, Germany).

### 2.7. Statistical Analysis 

Results (mean ± standard deviation; n = 3) were assessed using one-way analysis of variance and the Tukey–Kramer honestly significant difference (HSD) test with *p* < 0.05 considered to represent statistical significance. All statistical analyses were performed using SPSS 22.0 (SPSS Inc., Chicago, IL, USA).

## 3. Results and Discussion

### 3.1. Chemical Structure Elucidation of Compound ***1***–***7*** from CLF

CLFs were extracted in 80% (v/v) aqueous MeOH, and the condensed extracts were partitioned into CLFE, CLFB, and CLFW. Repeated column chromatography (CC) of the CLFE and CLFB gave seven flavanones, compounds **1**–**7**. Comparing the 1D-, and 2D-NMR and fab mass spectrometry (FM) data with reported values allowed us to identify two known compounds, 8-methoxybutin (**1**) [14] and flavanomarein (**3**) [15]. The other five compounds are newly reported here.

Compound **2**, a red amorphous powder (AP), indicated UV absorption characteristics (UVAC) at 254 and 365 nm and an orange color on thin layer chromatography (TLC) by 10% H_2_SO_4_ spraying and heating. The molecular formula (MF) was established as C_17_H_16_O_6_ from the molecular ion peak (MIP) [M + H]^+^ m/z 317.1028 (calcd. 317.1025 for C_17_H_17_O_6_) in the positive high resolution-FM (HR-FM). The infrared spectrum (IRS, cm^−1^) suggested the presence of aromatic double bond (1579), conjugated ketone (1637), and hydroxyl (3427). The ^1^H-NMR (PMR) exhibited the signals of two aromatic methines (chemical shift, integration, coupling pattern, J in Hz, proton number; δ_H_ 6.48, 1H, d, 8.8, H6; 7.47, 1H, d, 8.8, H5) because of a 1,2,3,4-tetrasubstituted benzene moiety and three aromatic methines (δ_H_ 6.94, 2H, br. s, H4′, H6′; δ_H_ 7.00, 1H, br. s, H2′) due to a 1,3,5-trisubstituted benzene moiety. In the oxygen region, one oxygenated methine proton (δ_H_ 5.37, 1H, dd, 12.6, 2.8, H-2) and two methoxy proton (δ_H_ 3.79, 3H, s; δ_H_ 3.86, 3H, s) signals were observed. In the aliphatic region, we observed two proton signals showing germinal coupling (J=17.0 Hz) (δ_H_ 2.71, 1H, dd, 17.0, 2.8, H3b; δ_H_ 2.98, 1H, dd, 17.0, 12.6, H3a) due to a methylene in the cyclohexane moiety. The PMR data predicted that compound **2** was a flavanone containing two hydroxy and two methoxy groups. The ^13^C-NMR (CMR) data showed 17 carbon signals including two methoxy groups (δ_C_ 56.1, 5′-OCH_3_; δ_C_ 61.3, 8-OCH_3_), confirming compound **2** to be a flavanone. The other carbon signals were derived from one ketone (δ_C_ 193.1, C4), five oxygenated olefin quaternaries (δ_C_ 137.2, C8; δ_C_ 147.8, C3′; δ_C_ 149.3, C5′; δ_C_ 159.8, C7; δ_C_ 161.0, C9), two olefin quaternaries (δ_C_ 114.2, C10; δ_C_ 133.6, C1′), five olefin methines (δ_C_ 112.4, C6; δ_C_ 112.6, C4′; δ_C_ 114.5, C2′; δ_C_ 118.8, C6′; δ_C_ 120.9, C5), one oxygenated methine (δ_C_ 81.0, C2), and one methylene (δ_C_ 44.8, C3). Since the circular dichroism (CD) spectrum of compound **2** displayed a positive cotton effect (CE) at 335 nm and a negative CE at 290 nm, the absolute configuration of C-2 was assigned as S configuration [20]. The gradient heteronuclear multiple bond coherence (gHMBC) spectrum revealed that two methoxy proton signals (δ_H_ 3.81, 3.75] correlated with the oxygenated olefin quaternary carbon signals (δ_C_ 149.3, C5′; δ_C_ 136.9, C8), suggesting that the methoxy groups are attached at the C-5′ and C-8 positions. Taken together, compound **2** was identified as a (2S)-7,3′-dihydroxy-8,5′-dimethoxyflavanone, a new compound, named coreolanceolin A.

Compound **4**, a red AP, indicated UVAC at 254 and 365 nm and a yellow color on TLC by 10% H_2_SO_4_ spraying and alcohol lamp heating. The MF was established as C_22_H_24_O_11_ from the MIP [M + H]^+^ m/z 465.1395 (calcd. 465.1396 for C_22_H_25_O_11_) in the positive HR-FM. The IRS (cm^−1^) suggested the presence of aromatic double bond (1580), conjugated ketone (1634), and hydroxyl (3490). The PMR and CMR of compound **4** were similar to those of compound **1**, except for signals indicating an additional monosaccharide moiety. The PMR signals of the monosaccharide included a hemiacetal (δ_H_ 5.68, d, 7.0, H-1″), four oxygenated methines (δ_H_ 4.13, 1H, overlapped), H3″; δ_H_ 4.25, 1H, OV, H4″; δ_H_ 4.30, 1H, OV, H5″; δ_H_ 4.34, 1H, OV, H2″), and one oxygenated methylene (δ_H_ 4.14, 1H, OV, H6″b; δ_H_ 4.50, 1H, br. d, 12.0, H6″a). The chemical shifts in the CMR signals were due to a hemiacetal (δ_C_ 101.8, C1″), four oxygenated methines (δ_C_ 70.9, C4″; 74.5, C2″; 77.9, C5″; 78.7, C3″), one oxygenated methylene (δ_C_ 62.1, C6″), and the coupling constant of the anomer proton signal (*J* = 7.0 Hz) revealed that the monosaccharide was a β-glucopyranose. In the gHMBC spectrum, the anomer proton signal (δ_H_ 5.70, H1″) showed a cross peak with the oxygenated olefin quaternary carbon signal (δ_C_ 157.3, C7), and the methoxy proton signal (δ_H_ 3.87, 3H, s) showed a cross peak with the oxygenated olefin quaternary carbon signal (δ_C_ 138.4, C8). Since the CD spectrum of compound **4** displayed a positive CE at 399 nm and a negative CE at 290 nm, the absolute configuration of C-2 was assigned as S configuration [20]. Consequently, compound **4** was identified as (2S)-3′,4′-dihydroxy-8-methoxyflavanone 7-O-β-D-glucopyranoside, named coreolanceolin B.

Compound **5**, a red AP, indicated UVAC at 254 and 365 nm and a yellow color on TLC by 10% H_2_SO_4_ spraying and alcohol lamp heating. The MF was established as C_22_H_24_O_11_ from the MIP [M – H]^–^ m/z 463.1238 (calcd. 463.1240 for C_22_H_23_O_11_) in the negative HR-FM. The IRS (cm^−1^) suggested the presence of aromatic double bond (1580), conjugated ketone (1634), and hydroxyl (3489). The PMR and CMR of compound **5** were similar to those of compound **3**, except for the signals of one additional methoxy group (δ_H_ 3.85, 3H, s; δ_C_ 56.4, 3′-OCH_3_). In the gHMBC spectrum, the methoxy proton signal (δ_H_ 3.85) displayed a cross peak with the oxygenated olefin quaternary carbon signal (δ_C_ 148.1, C3′), suggesting that the methoxy group was at the 3′ position. Since the CD spectrum of compound **5** displayed a positive CE at 387 nm and a negative CE at 287 nm, the absolute configuration of the chiral carbon C-2 was assigned as S configuration [20]. Accordingly, compound **5** was identified as (2S)-8,4′-dihydroxy-3′-methoxyflavanone 7-O-β-D-glucopyranoside, named coreolanceolin C.

Compound **6**, a red AP, indicated UVAC at 254 and 365 nm and a yellow color on TLC by 10% H_2_SO_4_ spraying and alcohol lamp heating. The MF was established as C_23_H_26_O_11_ from the MIP [M – H]^–^ m/z 477.1395 (calcd. 477.1396 for C_23_H_25_O_11_) in the negative HR-FM. The IRS (cm^−1^) suggested the presence of aromatic double bond (1590), conjugated ketone (1640), and hydroxyl (3500). The PMR and CMR of compound **6** were similar to those of compound **4** with the exception of one additional methoxy group signal (δ_H_ 3.86, s; δ_C_ 56.2, 4′-OCH_3_). In the gHMBC spectrum, two methoxy proton signals (δ_H_ 3.86, 3.83) displayed cross peaks with the two oxygenated olefin quaternary carbon signals (δ_C_ 149.3, C4′; δ_C_ 138.1, C8, respectively), suggesting that the methoxy groups are attached at the C-4′ and C-8 positions. The CD spectrum of compound **6** displayed a positive CE at 386 nm and a negative CE at 274 nm, so compound 6 was assigned the absolute configuration of 2S [20]. Therefore, compound **6** was established as (2S)-3′-hydroxy-8,4′-dimethoxyflavanone 7-O-β-D-glucopyranoside, named coreolanceolin D.

**Figure 2:**
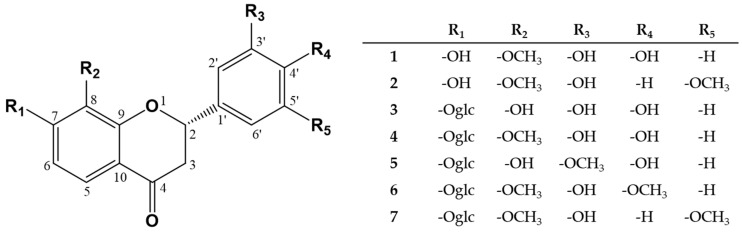
Structures of compound **1**−**7** from the flowers of *Coreopsis lanceolata*. glc: *β*-D-glucopyranosyl.

Compound **7**, a red AP, displayed UVAC at 254 and 365 nm and an orange color on TLC by 10% H_2_SO_4_ spraying and alcohol lamp heating. The MF was established as C_23_H_26_O_11_ from the MIP [M + Na]^+^ m/z 501.1374 (calcd. 501.1372 for C_23_H_26_O_11_Na) in the positive HR-FM. The IRS suggested the presence of hydroxyl group (3502 cm^−1^), conjugated ketone group (1638 cm^−1^), and an aromatic double bonds (1588 cm^−1^). The PMR and CMR of compound **7** were similar to those of compound **2**, with the exception of the signals from one additional monosaccharide. The PMR signals of the monosaccharide included a hemiacetal (δ_H_ 5.04, d, 7.2, H-1″), four oxygenated methines (δ_H_ 3.36, 1H, OV, H3″; δ_H_ 3.41, 1H, OV, H4″; δ_H_ 3.47, 1H, OV, H5″; δ_H_ 3.53, 1H, OV, H2″), and one oxygenated methylene (δ_H_ 3.69, 1H, dd, 12.8, 5.6, H6″b; δ_H_ 3.89, 1H, br. d, 12.8, H6″a). The chemical shifts of the signals in the CMR spectrum, a hemiacetal (δ_C_ 101.9, C1″), four oxygenated methines (δ_C_ 71.2, C4″; 74.8, C2″; 78.0, C5″; 78.3, C3″), and one oxygenated methylene (δ_C_ 62.4, C6″) indicated the monosaccharide to be a β-glucopyranose, and the anomer proton coupling constant (*J* = 7.2 Hz) confirmed the anomer hydroxy to have a β-configuration. In the gHMBC spectrum, the anomer proton signal (δ_H_ 5.04, H1″) displayed a cross peak with the oxygenated olefin quaternary carbon signal (δ_C_ 158.0, C7), suggesting that the β-D-glucopyranose is attached at the C-7 position. The CD spectrum of compound **7** displayed a positive CE at 391 nm and a negative CE at 277 nm, so compound **7** was assigned the absolute configuration 2S [20]. Therefore, **7** was revealed as (2S)-3′-hydroxy-8,5′-dimethoxyflavanone 7-O-β-D-glucopyranoside, named coreolanceolin E. Figure 2 showed the chemical structure of compound **1**–**7** from CLF.

### 3.2. Quantitative Analysis of Compound ***1***–***7*** in the Extracts from CLF

**Figure 3:**
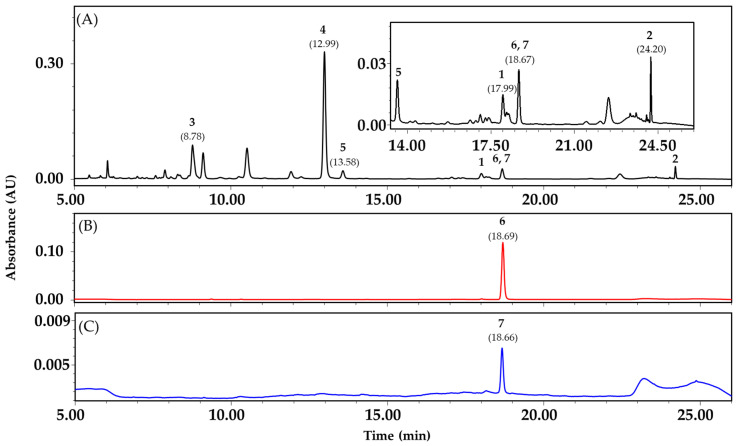
HPLC chromatograms. (**A**) Extract from *Coreopsis lanceolata* flowers; (**B**) flavanone **6**; (**C**) flavanone **7.**

Quantitative analysis of compound **1**–**7** in the extract was conducted. Gradient elution using water and acetonitrile was performed, using a Poroshell 120 SB-C_18_ column. Most peaks were eluted within 26 min and detected at 360 nm. The peaks that appeared at 8.78, 12.99, 13.58, 17.99, 18.67, 18.67, and 24.20 min were identified as flavanomarein (**3**), coreolanceolin B (**4**), coreolanceolin C (**5**), 8-methoxybutin (**1**), coreolanceolin D (**6**), coreolanceolin E (**7**), and coreolanceolin A (**2**), respectively, by comparing their retention times with those of standard compounds (Figure 3). This method was reliable since the r^2^ values of the regression curves were all bigger than 0.99. The contents of compound **1**–**7** in CLF are presented in Table 3.

### 3.3. Radical Scavenging Activities of Compound ***1***–***7*** Using DPPH and ABTS Assays

The radical scavenging activities of compound **1**–**7** in the DPPH and ABTS radical assays were presented in Table 4. In the DPPH radical scavenging assay, compound **1** displayed the highest scavenging potential and compounds **2**, **3**, and **4** showed significant activity, whereas compounds **5**–**7** showed low antioxidant activity. In the ABTS assay, compounds **1** and **2** exhibited slightly higher antioxidant activity than that of vitamin C. These results indicate unambiguous structure-activity relationships. The aglycone compounds **1** and **2** showed higher activity than the monoglucoside compounds **3**–**7**. The sugar moiety in flavanones reduces antioxidant activity because the free 7-hydroxy group in the A-ring is the key structure enabling antioxidant activity, which was clearly proven for naringenin and naringenin 7-rutinoside [21,22]. The DPPH scavenging activity of compound **1** was higher than that of compound **2**, and the activities of compounds **3** and **4** were higher than those of compounds **5**–**7.** Notably, compound **1**, **3**, and **4** contain a 3,4-dihydroxyphenyl group, a catechol structure. The presence of −OH groups in the aromatic ring of flavonoids is considered essential for radical scavenging activity, and a catechol moiety in the B-ring confers high stability to the phenoxyl radical via electron delocalization after hydrogen dissociation [23,24],. The DPPH scavenging action of compound **3** was higher than that of compound **4**, and the action of compound **5** was higher than of compounds **6** and **7** because the 8-hydroxy or/and 4′-hydroxy groups of compounds **6** and **7** were protected. The influence of flavanone glycosides on antioxidant activity was reported as follows; 8-methoxyl or/and 4′-methoxyl groups were consistently less active than corresponding compounds with a free hydroxyl group [22]. Therefore, the catechol group at the B-ring, the free hydroxyl group of C-8 or C-4′, and the absence of an attached sugar are the key factors that contribute to the potency of the radical scavenging capacity.

### 3.4. Inhibition Effects of Compound ***1***–***7*** on Intracellular OS in PC-12, Caco-2, and RAW 264.7 Cells

**Figure 4:**
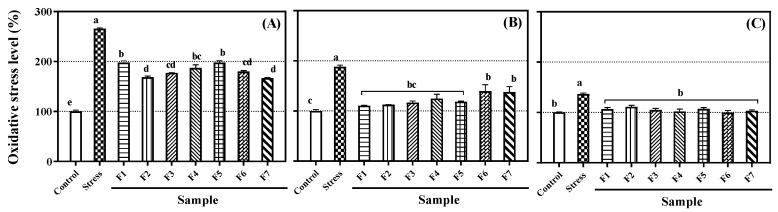
Recovery effects of flavanones **1**–**7** (F1~F7) on intracellular oxidative stress in: (**A**) Caco-2 epithelial cells; (**B**) RAW 264.7 macrophages; (**C**) PC-12 neurons. Lowercase letters on the bars indicate significant differences according to the Tukey–Kramer HSD test (*p* < 0.05).

ROS are reducing metabolites of oxygen which are generated by metabolic processes or external elements in normal cells in the body, and most of them have an unstable state that allows them to lose or obtain electrons and a stable state. These properties are known to cause OS in DNA and cell membranes in vivo, causing damage and various diseases, inflammation, and aging [25,26]. DCFH-DA is a representative material for measuring ROS in cells. It can freely pass through the cell membrane and is deacetylated with non-fluorescent DCFH when the acetate group is removed by intracellular esterase. Deacetylated DCFH is oxidized by ROS, such as H_2_O_2_, and thus becomes a strong fluorescent DCF. The isolated compounds from CLF inhibited intracellular ROS in Caco-2 colon epithelial, PC-12 neuronal, and RAW 264.7 macrophage cells (Figure 4). ROS levels in all three cell lines were increased by OS (200 μM H_2_O_2_) compared to the levels in control cells (Caco-2: 265.7%, RAW 264.7: 188.0%, and PC-12: 136.6%). After treating the cells with isolated flavanones (10 μM), we confirmed that all the flavanones significantly lowered the ROS-induced stress. However, the reduction rates varied by cell line. In Caco-2 cells, flavanones **2** and **7** lowered ROS stress levels; flavanones **1**−**5** were efficacious in RAW 264.7 cells, and all flavanones lowered ROS stress levels in PC-12 cells. These results also exhibited unambiguous structure-activity relationships. In the Caco-2 cells, flavanones **2** and **7** were the most efficacious, indicating that the catechol structure in the B-ring hindered ROS effects. Flavanone **4**, which has an additional glucose compared with flavanone **1**, showed a little more efficacy than flavanone **1**. In the RAW 264.7 cells, flavanones **2** and **7**, which are aglycones, were more effective than the glycosides. Among flavanones **3**, **4** and **6**, the more methoxy groups in the flavanones, the lower recovery effects. Therefore, flavanones **7** and **8** showed the lowest recovery effects because they have attached sugars and two methoxy groups. In PC-12 cells, all the compounds restored intracellular OS to the control level, and no deviations caused by differences in structure, such as −OH, −glucose, and −OCH_3_, were observed. Flavonoids have different absorption and transmission rates in cells depending on their structure [27,28]. Therefore, compounds with different functional groups are thought to have different degrees of access and absorption in different types of cells. In addition, the flavonoids have different antioxidant properties depending on the number or position of their OH and various other structural characteristics, such as double bonds, protection of OH, and number of sugars [29]. For this reason, even though the aglycone compounds were the strongest antioxidants, flavanones **3**–**7** had a better ability to reduce OS in cells than flavanones **1** and **2**. Therefore, the ability to reduce the OS in a particular cell depends on a combination of absorption, permeability, and molecular antioxidant capacity.

### 3.5. Inhibitory Effects of Flavanones ***1***–***7*** on NO Production in RAW 264.7 Cells

**Figure 5:**
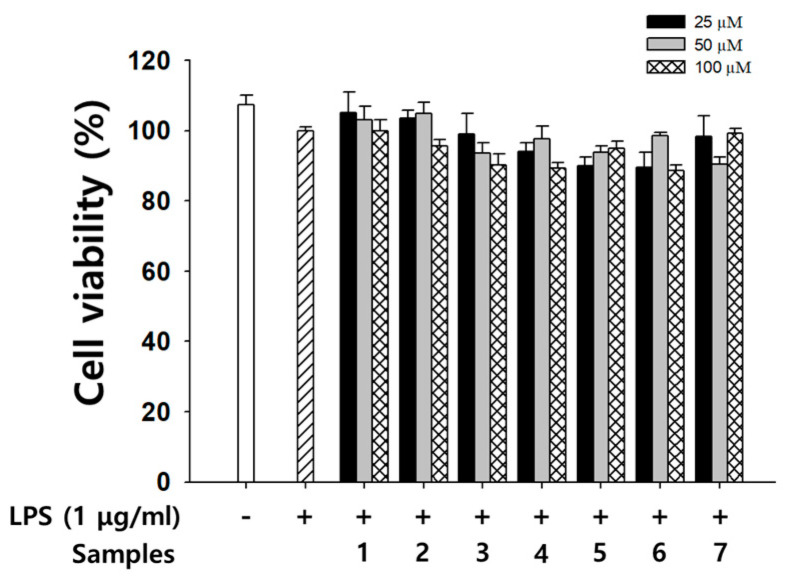
Effects of flavanones on cell viability of RAW 264.7cells. The experiments were done in triplicates.

NO, a signaling molecule that plays a key role in the pathogenesis of inflammation, is produced by mouse macrophages in response to LPS [30]. NO is thought to cause several chronic inflammatory diseases such as inflammatory bowel disease and arthritis as well as certain autoimmune disorders [31,32,33]. Therefore, we evaluated the effects of flavanones **1**–**7** on NO production in RAW 264.7 cells at dose of none cytotoxic effects. All compounds showed no cytotoxicity lower than 100 μM. (Figure 5).

**Figure 6:**
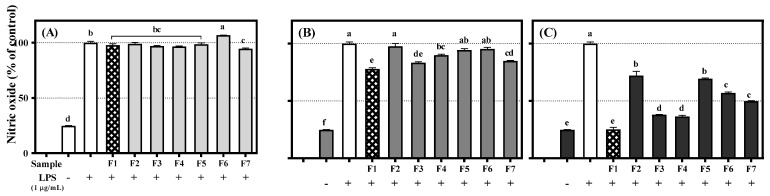
Inhibition effect of flavanones **1**−**7** on nitric oxide production in RAW 264.7 cells: (**A**) 25 μM; (**B**) 50 μM; (**C**) 100 μM. Lowercase letters on the bars indicate significant differences according to the Tukey–Kramer HSD test (*p* < 0.05).

Figure 6 showed that LPS treatment induced an increase in NO production. NO is synthesized by endothelial nitric oxide synthase. All seven flavanones significantly reduced NO production at 100 μM. In particular, flavanone 1 inhibited NO production to the control level (Figure 6C), similarly to 10 μM AMT, which is commonly used as a positive control (83% inhibited to control) [34]. Flavanones **3**, **4**, and **7** inhibited NO production more than flavanones **2** and **5**. Therefore, flavanones **1**, **3**, **4**, and **7** have potential as pro-inflammatory inhibition materials. A distinct structure-activity relationship was also observed in this case. Flavanones **1**, **3**, and **4** were notably effective in inhibiting NO production because of the catechol moieties in their B-rings. The catechol groups inhibit NO production through the inhibition of LPS signaling and direct scavenging of NO [35].

### 3.6. Inhibitory Effects of Flavanone ***1***–***7*** on Expression of iNOS and COX-2 in RAW 264.7 Cells

**Figure 7:**
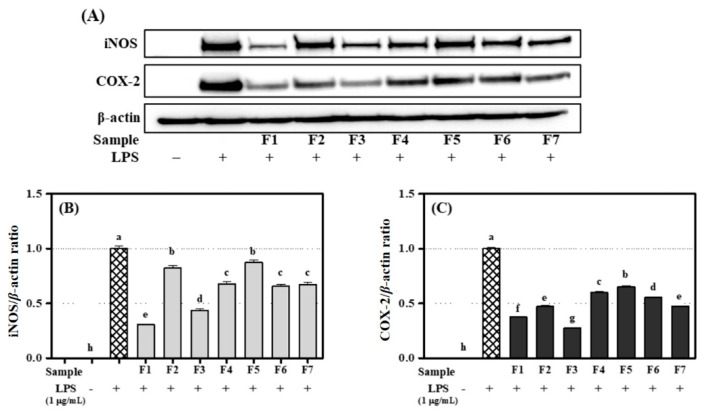
Inhibition effect of flavanones **1**−**7** (100 μM) on the expression of iNOS and COX-2 in RAW 264.7 cells: (**A**) Immunoblotting method; (**B**) iNOS/*β*-actin ratio; (**C**) COX-2/*β*-actin ratio. Lowercase letters on the bars indicate significant differences according to the Tukey–Kramer HSD test (*p* < 0.05).

When inflammation occurs in response to activating signals such as LPS and/or cytokines, iNOS produces NO in macrophages [13,33,36]. In addition, the level of COX-2, a pro-inflammatory enzyme, can also indicate the extent of inflammation [13,36,37]. The effect of the flavanones on iNOS and COX-2 expression was evaluated using western blot experiments. β-Actin, a housekeeping gene that is expressed at a stable level in varying cellular conditions, was used as an internal loading control for normalization. Flavanones **1**, **3**, **4**, **6**, and **7** inhibited iNOS expression in RAW 264.7 cells relatively strongly. (Figure 7) This result showed the same tendency as their ability to inhibit NO production. A previous study also reported that NO and iNOS share generation mechanisms [38]. In addition, flavanones **1**, **3**, **4**, **6**, and **7** are thought to not only inhibit NO production, but also affect the gene promoter that produces iNOS. However, we did not determine the detailed for that effect in this study. The inflammatory enzymes iNOS and COX-2 are regulated by the same factors and are often expressed together in inflammatory reactions. COX-2 is undetectable in most tissues but is expressed during inflammatory responses [39]. All seven flavanones markedly inhibited COX-2 expression in RAW 264.7 cells. Flavanones **1** and **3** showed the highest inhibitory effects on the expression of iNOS and COX-2 because of the presence of catechol groups in the B-ring [35].

## 4. Conclusions

New anti-inflammatory flavanones from CLF were identified and quantified. Five new flavanones, coreolanceolins A–E, along with two previously known flavanones, were isolated through repeated CC using silica gel, octadecyl silica gel, and Sephadex LH-20 resins, and their chemical structures were determined without ambiguity based on the intensive analysis of 1D-, 2D-NMR, UV, IR, MS, and CD data. Most flavanones showed significant radical scavenging activities, with flavanones **1** and **2** showing the highest radical scavenging activities. All seven flavanones showed potential to be powerful antioxidants by reducing OS in Caco-2 intestinal epithelial cells, RAW 264.7 macrophage cells, and PC-12 neuron cells. Moreover, these antioxidants suppressed pro-inflammatory enzymes iNOS and COX-2 in RAW 264.7 macrophages. We have also provided a reasonable explanation for the structure-activity relationship in our results. The catechol group at the B-ring, a free hydroxyl moiety at C-8 or C-4′, and the absence of an attached sugar are key factors that contribute to radical scavenging capacity, the suppression of ROS production, inhibition of NO production, and inhibition of iNOS and COX-2 expression. Although the exact mechanisms, such as the expression of specific proteins at the molecular level, remain to be determined, these results indicate that flavanones obtained from CLF could be used as potential antioxidant and anti-inflammatory agents.

## Figures and Tables

**Table 1:** The ^1^H-NMR data for compound **2** and **4**–**7**. δH in ppm, *J* in Hz.

No.	δ_H_, Coupling Pattern, *J* in Hz				
	2 ^1^	4 ^2^	5 ^1^	6 ^1^	7 ^1^
**2**	5.37, dd, 12.6, 2.8	5.56, dd, 12.0, 2.4	5.43, br. d, 12.0	5.43, dd, 12.8, 2.8	5.45, dd, 12.4, 2.4
**3**	2.98, dd, 17.0, 12.6 2.71, dd, 17.0, 2.8	3.23, dd, 16.8, 12.0 3.01, dd, 16.8, 2.4	3.14, dd, 17.6, 12.0 2.77, br. d, 17.6	3.05, dd, 17.0, 12.8 2.79, dd, 17.0, 2.8	3.07, dd, 16.8, 12.4 2.81, dd, 16.8, 2.4
**5**	7.47, d, 8.8	7.84, d, 9.2	7.34, d, 8.8	7.84, d, 9.2	7.60, d, 8.8
**6**	6.48, d, 8.8	7.24, d, 9.2	6.89, d, 8.8	6.81, d, 9.2	6.92, d, 8.8
**2′**	7.00, br. s	7.53, d, 1.6	7.13, br. s	7.23, br. s	7.00, br. s
**4′**	6.94, br. s	-	-	-	6.94, br. s
**5′**	-	7.59, d, 8.0	6.79, d, 8.0	6.89, d, 8.0	-
**6′**	6.94, br. s	7.09, dd, 8.0, 1.6	6.93, br. d, 8.0	6.93, br. d, 8.0	6.94, br. s
**8-OCH_3_**	3.79, s	3.87, s	-	3.83, s	3.84, s
**3′-OCH_3_**	-	-	3.85, s	-	-
**4′-OCH_3_**	-	-	-	3.86, s	-
**5′-OCH_3_**	3.86, s	-	-	-	3.86, s
**1″**	-	5.68, d, 7.0	4.93, d, 7.2	5.06, d, 8.0	5.04, d, 7.2
**2″**	-	4.34 ^#3^	3.52 ^#^	3.53 ^#^	3.53 ^#^
**3″**	-	4.13 ^#^	3.38 ^#^	3.36 ^#^	3.36 ^#^
**4″**	-	4.25 ^#^	3.41 ^#^	3.42 ^#^	3.41 ^#^
**5″**	-	4.30 ^#^	3.50 ^#^	3.47 ^#^	3.47 ^#^
**6″**	-	4.50, br. d, 12.0 4.14 ^#^	3.89, br. d, 12.0 3.81, dd, 12.0, 5.6	3.89, br. d, 12.0 3.70, dd, 12.0, 5.6	3.89, br. d, 12.8 3.69, dd, 12.8, 5.6

^1^ H-NMR spectrum was measured in CD_3_OD at 400 MHz. ^2^ H-NMR spectrum was measured in pyridine-*d*_5_ at 400 MHz. ^3 #^, overlapped.

**Table 2:** The ^13^C-NMR data for compound **2** and **4**–**7**. δ_C_ in ppm.

No.	δ_C_				
	2 ^1^	4 ^2^	5 ^1^	6 ^1^	7 ^1^
**2**	81.0	80.8	81.6	81.4	81.2
**3**	44.8	44.4	44.9	45.0	44.9
**4**	193.1	191.3	194.2	194.2	193.4
**5**	123.9	122.7	118.7	122.3	123.4
**6**	113.4	110.0	110.7	107.6	110.9
**7**	159.8	157.3	152.9	157.3	158.0
**8**	137.2	138.4	133.4	138.1	138.0
**9**	161.0	156.3	152.4	159.0	160.6
**10**	114.2	117.6	115.9	117.6	118.2
**1′**	133.6	130.9	130.8	132.7	133.1
**2′**	113.4	115.0	111.6	114.6	114.6
**3′**	147.8	147.1	148.1	147.8	147.8
**4′**	112.7	147.5	149.1	149.3	112.6
**5′**	149.3	116.4	116.0	115.2	149.4
**6′**	118.8	118.6	120.6	119.1	118.2
**1″**	-	101.8	102.9	101.7	101.9
**2″**	-	74.5	74.7	74.7	74.8
**3″**	-	78.7	78.4	78.3	78.3
**4″**	-	70.9	71.2	70.9	71.2
**5″**	-	77.9	77.5	77.6	78.0
**6″**	-	62.1	62.7	62.7	62.4
**8-OCH_3_**	61.1	60.9	-	61.3	61.7
**3′-OCH_3_**	-	-	56.4	-	-
**4′-OCH_3_**	-	-	-	56.2	-
**5′-OCH_3_**	56.4	-	-	-	56.7

^1 13^C-NMR spectrum was measured in CD_3_OD at 100 MHz. ^2 13^C-NMR spectrum was measured in pyridine-*d*_5_^b)^ at 100 MHz.

**Table 3:** Concentration (mg/g) of compound **1**–**7** in *Coreopsis lanceolata* flowers using reversed-phase high-performance liquid chromatography

Compound	Retention Time (min)	Regression Equation	*r* ^2^	Concentration
**1**	17.99	y = 5008x + 25457	0.999	5.4 ± 0.3
**2**	24.20	y = 6074.7x + 17458	0.999	3.9 ± 1.1
**3**	8.78	y = 20438x + 40392	0.999	11.3 ± 0.1
**4**	12.99	y = 19111x + 63019	0.998	38.8 ± 0.3
**5**	13.58	y = 11101x + 2790.1	0.999	5.2 ± 0.2
**6**	18.67	y = 19952x + 13540	0.999	2.5 ± 0.2
**7**	18.67	y = 12915x − 10826	0.999	0.8 ± 0.1

**Table 4:** Radical-scavenging activity of flavanones 1−7 from *Coreopsis lanceolata* flowers in the DPPH and ABTS radicals assays.

Compound	DPPH Radical (mg VCE ^1^/1000 mg)	ABTS Radical (mg VCE/1000 mg) ^1^
**1**	104.3 ± 1.9 ^a2^	1278.6 ± 26.8 ^a^
**2**	85.0 ± 4.0 ^b^	1095.7 ± 0.1 ^b^
**3**	76.5 ± 3.2 ^c^	624.8 ± 3.1 ^c^
**4**	68.8 ± 0.8 ^d^	610.8 ± 3.9 ^c^
**5**	29.0 ± 0.1 ^e^	519.2 ± 0.7 ^d^
**6**	27.1 ± 1.3 ^e^	456.5 ± 0.5 ^e^
**7**	20.5 ± 0.3 ^f^	325.6 ± 0.2 ^f^

^1^ VCE vitamin C equivalent (VCE). ^2^ Data are presented as the mean ± standard deviation (*n* = 3). Means with different superscripts in the same column differ significantly different by Tukey-Kramer’s HSD test (*p* < 0.05).

## Supplementary Materials

The following are available online at https://www.mdpi.com/2076-3921/9/6/539/s1. The isolation methods of compound **1**–**7,** PMR, CMR, HR-FABMS data of **2** and **4**–**7** are available as supporting information.

## Abbreviations

AAPH	2,2′-azino-bis(2-amidinopropane) dihydrochloride
ABTS	2,2′-azino-bis(3-ethylbenzothiazoline-6-sulfonic acid
AMT	amino-5,6-dihydro-6-methyl-4H-1,3-thiazine hydrochloride
AP	amorphous powder
CC	column chromatography
CD	circular dichroism
CE	cotton effect
CLF	Coreopsis lanceolata flowers
CLFE	*Coreopsis lanceolata* flowers ethyl acetate fraction
CLFB	*Coreopsis lanceolata* flowers *n*-butanol fraction
CLFW	*Coreopsis lanceolata* flowers water fraction
CMR	carbon-13 nuclear magnetic resonance (^13^C-NMR)
CMRM100	^13^C-NMR (100 MHz, CD_3_OD, δ_C_)
COX-2	cyclooxygenase-2
DCFH-DA	2′,7′-dichlorofluorescein diacetate
DMEM	Dulbecco’s modified Eagle’s medium
DPPH	2,2-diphenyl-1-picrylhydrazyl
DW	dry weight
EtOAc	ethyl acetate
EV/TV	elution volume/total volume
FM	Fast atom bombardment mass spectroscopy (FAB-MS)
FBS	fetal bovine serum
frs.	fractions
gHMBC	gradient heteronuclear multiple bond coherence
H_2_O_2_	hydrogen peroxide
HPLC	high-performance liquid chromatography
HR	high resolution
HSD	honestly significant difference
iNOS	inducible nitric oxide synthase
IRS	infrared spectrum
MeOH	methanol
MF	molecular formula
MIP	molecular ion peak
MTT	3-(4,5-dimethylthiazol-2-yl)-2,5-diphenyl-tetrazolium bromide
NBT	nitro blue tetrazolium
*n*-BuOH	*n*-butanol
NMR	nuclear magnetic resonance
NO	nitric oxide
OV	overlapped
PMR	proton-1 nuclear magnetic resonance (^1^H-NMR)
PMRM400	^1^H-NMR (400 MHz, CD_3_OD, δ_H_)
ROS	reactive oxygen species
RPMI	1640 Roswell Park Memorial Institute 1640
SiO_2_	silica gel
SO	superoxide
ODS	octadecyl silica gel
OS	oxidative stress
PBS	phosphate-buffered saline
TLC	thin layer chromatography
UV	ultraviolet
UVAC	UV absorption characteristics
1D-NMR	one-dimensional (1D) NMR
2D-NMR	Two-dimensional (2D) NMR
